# Test–retest reliability of an infectious disease questionnaire and evaluation of self-assessed vulnerability to infections: Findings of Pretest 2 of the German National Cohort

**DOI:** 10.1007/s00103-014-2045-x

**Published:** 2014-10-08

**Authors:** S. Castell, M.K. Akmatov, N. Obi, D. Flesh-Janys, A. Nieters, Y. Kemmling, F. Pessler, G. Krause

**Affiliations:** 1 Department for Epidemiology, Helmholtz Centre for Infection Research, Inhoffenstraße 7, 38124 Braunschweig, Germany; 2 Department of Cancer Epidemiology, Clinical Cancer Registry, University Cancer Center Hamburg (UCCH), Hamburg, Germany; 3 Centre for Chronic Immunodeficiency, Freiburg, Germany; 4 TWINCORE Centre for Experimental and Clinical Infection Research, Hanover, Germany; 5 Hanover Medical School, Hanover, Germany

**Keywords:** Infections, Infectious diseases, Infectious disease questionnaire, Test–retest reliability, German National Cohort (GNC), Infektionen, Infektionskrankheiten, Fragebogen zu Infektionskrankheiten, Test-Retest-Reliabilität, Nationale Kohorte (NAKO)

## Abstract

**Introduction/objectives:**

Large scale population-based studies focusing on infectious diseases are scarce. This may be explained by methodological obstacles concerning ascertainment of data on infectious diseases requiring, e.g. collection of data on relatively short-termed symptoms and/or collection of biosamples for pathogen identification during a narrow time window. In the German National Cohort (GNC), a novel self-administered questionnaire will be used in addition to biosampling to collect data on selected infectious diseases and symptoms. The aim of this study was to evaluate in Pretest 2 of the GNC newly added items on self-assessed vulnerability to several infectious diseases and to assess test–retest reliability of the questionnaire.

**Methods:**

The study was conducted in two study centres (Hamburg and Hanover) during Pretest 2 of the GNC. A self-administered paper questionnaire was applied. In Hamburg, participants were asked to fill in the questionnaire during their regular visit at the study centre. For test–retest reliability, participants in Hanover filled in the same questionnaire at home twice. To evaluate agreement, item-related percentage agreement and kappa (κ) were calculated. In addition, we computed Bennet’s S and Krippendorf’s alpha (α). Items on self-assessed vulnerability to infections were evaluated by comparing them with the corresponding self-reported frequency of infections. An explanatory factor analysis was applied to construct the scores of self-reported infection frequency and self-assessed vulnerability to infections.

**Results:**

The evaluation of the internal consistency of the five-item instrument of self-assessed vulnerability to infections resulted in a Cronbach’s α of 0.78. The factor analysis yielded evidence of one factor. The factor was divided into three groups (lowest quintile classified as “less prone to infections” compared to peers; second, middle and fourth quintiles classified as “similarly prone to infections” and highest quintile classified as “more prone to infections”). Participants classified as “less prone to infections” reported fewer infections than participants classified as “more prone to infections”. Spearman’s correlation of the two scores (self-reported infection frequency and self-assessed vulnerability to infection) was 0.50 (*p* < 0.0001). For quantifying reliability, 88 participants with a median time of 8 days between filling in both questionnaires could be included in the analysis; for items sensitive to disease occurrence between both questionnaires only participants with no relevant disease in this time interval were included (*n* = 75). The weighted κ ranged between 0.65 and 0.87 for the items on infectious disease frequency in the last 12 months, for items on symptom frequency in the past 12 months between 0.77 and 0.90, and for items on vulnerability compared to peers between 0.68 and 0.76.

**Conclusion:**

A five-item instrument on self-assessed vulnerability to infections seems to be promising, but requires further evaluation. Overall, the questionnaire on self-reported infectious diseases used in Pretest 2 of the GNC is a moderately reliable instrument and, thus, can be applied in future studies on infectious diseases.

**Electronic supplementary material:**

The online version of this article (doi: 10.1007/s00103-014-2045-x) contains supplementary material, which is available to authorized users.

## Abstract (de)

**Einführung/Ziele:**

Groß angelegte populationsbasierte Studien, die Infektionskrankheiten zum Thema haben, sind selten. Dies könnte sich daraus erklären, dass die Erhebung von Daten zu Infektionskrankheiten methodisch schwierig ist. So ist es in diesem Zusammenhang z. B. erforderlich, Informationen zu nur relativ kurzzeitig auftretenden Symptomen zu erheben und/oder Bioproben zur Pathogenbestimmung zu gewinnen. In der Nationalen Kohorte (NAKO) wird zusätzlich zur Bioprobengewinnung ein neuer Fragebogen angewandt werden, um Daten zu ausgewählten Infektionskrankheiten und ihren Symptomen zu erfassen. Ziel der vorliegenden Studie war es, für den Pretest 2 der NAKO neu ergänzte Fragenbogenitems zur selbst eingeschätzten Anfälligkeit gegenüber verschiedenen Infektionskrankheiten zu evaluieren und die Test-Retest-Reliabilität des Fragebogens zu prüfen.

**Methoden:**

Die Studie wurde im Rahmen des Pretests 2 der NAKO in 2 Studienzentren durchgeführt (Hamburg und Hannover). Die Teilnehmer wurden gebeten, einen Papierfragebogen eigenständig auszufüllen. Die Teilnehmer in Hamburg füllten den Fragebogen im Rahmen der Untersuchung im Studienzentrum aus. Die Test-Retest-Reliabilität wurde bei Probanden in Hannover überprüft, indem sie den gleichen Fragebogen 2-mal zu Hause ausfüllten. Zur Bestimmung der Konkordanz wurden die itembezogene prozentuale Übereinstimmung und Kappa (κ) berechnet. Ergänzend berechneten wir Bennets S und Krippendorfs α. Die Items der selbst eingeschätzten Infektionsanfälligkeit wurden evaluiert, indem sie mit den korrespondierenden Items zu selbst berichteten Infektionshäufigkeiten verglichen wurden. Um Scores für selbst berichtete Infektionshäufigkeit und selbst eingeschätzte Infektanfälligkeit zu entwickeln, wurde eine explorative Faktorenanalyse angewendet.

**Ergebnisse:**

Die Evaluierung der internen Konsistenz des aus 5 Items bestehenden Instrumentes zur selbst eingeschätzten Infektanfälligkeit ergab ein Cronbachs α von 0,78. Durch die Faktorenanalyse wurde ein Faktor ermittelt. Der ermittelte Faktor wurde in 3 Gruppen unterteilt. Das unterste Quintil wurde als „weniger infektanfällig“, das zweite, dritte und vierte Quintil als „ähnlich infektanfällig“ und das oberste Quintil als „infektanfälliger“ im Vergleich zu Gleichaltrigen definiert. Probanden in der Gruppe „weniger infektanfällig“ berichteten weniger Infektionen als Teilnehmer, die als „infektanfälliger“ klassifiziert wurde. Die Korrelation (Spearman) der beiden Scores (selbst berichtete Infektionshäufigkeit und selbst eingeschätzte Infektionsanfälligkeit) ergab einen Wert von 0,50 (*p* < 0,0001). Um die Reliabilität zu quantifizieren, konnten 88 Teilnehmer mit einem medianen Zeitraum von 8 Tagen zwischen beiden Fragebögen für die Berechnungen berücksichtigt werden; Items, die vom Auftreten von Krankheiten zwischen dem Ausfüllen beider Fragebögen beeinflusst werden könnten, wurden nur unter Berücksichtigung von Teilnehmern ohne relevante Erkrankungen in diesem Zeitraum analysiert (*n* = 75). Das gewichtete κ bewegte sich zwischen 0,65 und 0,87 für Items zur Häufigkeit von Infektionskrankheiten in den vergangenen 12 Monaten, zwischen 0,77 und 0,90 für Items zur Symptomhäufigkeit in den vergangenen 12 Monaten und zwischen 0,68 und 0,76 für Items zur Infektanfälligkeit verglichen mit Gleichaltrigen.

**Schlussfolgerung:**

Das aus 5 Items bestehende Instrument zur selbst eingeschätzten Infektionsanfälligkeit scheint vielversprechend zu sein. Allerdings sollte es noch weitergehend validiert werden. Grundsätzlich ist der Fragebogen zu selbst berichteten Infektionskrankheiten, wie er im Pretest 2 der NAKO verwendet wurde, ein relativ reliables Instrument und kann daher in weiteren Studien zu Infektionskrankheiten angewendet werden.

Large scale population-based studies focusing on infectious diseases are scarce. This may be explained by methodological obstacles concerning infectious diseases. Many common infectious diseases are acute in nature, requiring an intensified collection of symptomatic data and/or collection of biosamples for pathogen identification. Apart from laboratory methods to detect past and/or current infections in the German National Cohort (GNC), a self-administered questionnaire will be used to collect additional data on selected infectious diseases. For this reason, a panel of experts involving infectious disease epidemiologists, medical doctors, microbiologists, public health specialists and others were invited to work on the development of an infectious disease questionnaire. An initial version of this questionnaire was applied during Pretest 1 of the GNC and is evaluated in a separate publication in this issue (Sievers et al.). We further modified the questionnaire for application in Pretest 2 by including additional questions (e.g. questions about self-assessed vulnerability to infections). Since this new instrument for susceptibility to infections has not been evaluated before, the aim of this study was to evaluate the respective items. Additionally, to further test the applicability of the questionnaire in the GNC we examined test–retest reliability of the questionnaire in Pretest 2.

## Methods

### Validation study

This part of the study was conducted in two study centres (Hamburg and Hanover) during the Pretest 2 phase of the GNC. In both study centres, a self-administered paper-based questionnaire was applied. In Hamburg, participants were asked to fill in the questionnaire on infectious diseases during their regular visit at the study centre. In Hanover, the first of the two questionnaires of the reliability study was used to evaluate the items on self-assessed vulnerability.

### Reliability study

Test–retest reliability was examined in Hanover only, i.e. by administering the infectious disease questionnaire on two separate occasions. The questionnaire was mailed twice to the participants. The second copy of the questionnaire was sent to the participants upon receipt of the first questionnaire in the study centre but no earlier than one week after sending the first copy. The second questionnaire contained a question about disease occurrence since completion of the first questionnaire in order to exclude those participants from analysis who experienced an intercurrent illness between both questionnaires which might influence the response. Participants who specified a recent relevant disease episode were excluded from the calculation of agreement for disease-based (IN1–IN7) and symptom-based (F1–F3) items as well as the question on antibiotic intake (A1).

### Infectious disease questionnaire

The complete questionnaire can be found online (see supplement).

#### Self-reported infections (IN1–7) and self-reported symptoms (F1–3)

The questionnaire contained seven questions asking about the frequency of upper and lower respiratory infections (U/LRT), gastrointestinal tract infections (GIT) and infections of the bladder or the kidney and skin infections in the last 12 months. There were six answer categories: no infection, once, twice, three-to-four times, more than four times and the category “I don’t know”. In addition, we asked about the frequencies of three syndrome-related outcomes (cough lasting over 4 weeks, fever and diarrhoea) in the last 12 months. Questions about outpatient treatment and hospitalisation (answer categories “yes”, “no”, “I don’t know”) were included for infections of the upper and lower respiratory tract (IN1a, IN1b, IN2a, IN2b) and of the gastrointestinal tract (IN3a, IN3b).

#### Self-assessed vulnerability to infections (IH1–5)

Self-assessed vulnerability to several selected infections (upper and lower respiratory tract infections, gastrointestinal tract infections, infections of the bladder or the kidney, and skin infections) was asked by questions like “Compared to individuals in my age group I have infections of e.g. the upper respiratory tract” … “far less frequently”, “less frequently”, “approximately equally frequently”, “more often” or “much more often”.

#### Other questions

In addition to these thematically connected item blocks we asked one question on antibiotic intake in the last 12 months (A1) with six answer categories (never, once, twice, three-to-four times, more than four times and “I don’t know”) and questions on influenza vaccination (see Schultze, Akmatov, Castell et al. in this issue). The reliability study used only the general item on this vaccination (V1).

### Definitions

Migration status was defined by either both parents not born in Germany, or one parent not born in Germany and interviewee not living in Germany since birth, or German not being native language [1]. Household net equivalent income per month was calculated from the original data using midpoint estimates of group levels; the highest group (≥ 8000 €) was set to 10,000 €. To account for household size, weighting was done according to [2]. School education was grouped as recommended by [3].

### Statistical analysis

#### Validation of self-assessed vulnerability

Internal consistency of the five-item instrument “self-assessed vulnerability to infections” (IH1–5) was examined by Chronbach’s alpha (α). An explanatory factor analysis with the Varimax rotation method was applied to construct the scores of self-reported frequency of infections (IN1–7) and self-assessed vulnerability to infections. The Kaiser–Meyer–Olkin measure was employed to check the sampling adequacy of both scores. Spearman’s correlation was used to examine the correlation between the two scores. Furthermore, the score of self-assessed infection vulnerability was divided into five groups of equal size (i.e. quintiles). We then grouped the second, third and fourth quintiles into one group, resulting in three groups (lowest quintile classified as “less prone to infections”, second, third and fourth quintiles as “similarly prone to infections” and highest quintile as “more prone to infections” compared to peers).

#### Reliability study

Participants who filled in both questionnaires within less than 5 days or more than 14 days were excluded from the reliability analysis so that, on one hand, memory effects would be reduced and, on the other hand, the stability of the attributes was approximately ensured [4]. Reliability was quantified using Cohen’s kappa (κ) [4]. For ordinal scales (answer categories: never/ once/ twice/ three-to-four times/ more than four times) linear weighted κ wasused to take magnitudes in disagreement into account. Linear weighting was chosen over quadratic weighting because it increases less with the number of categories [4]. The weighting matrix for weighted κ was calculated based on the formula $linear\text{ }weight=1-\frac{\left| i- \right.\left. j \right|}{k-1}\,$ where *i* and *j* are specific row and column categories and *k* is the overall number of categories [4]. If e.g. only 4 of 7 categories were used by the study population the matrix was modified to maintain the same weight of a given cell of the contingency table. In addition to the weighted κ, unweighted κ was calculated to account also for missing values (in one questionnaire) and the category “I don’t know”. In this case, answers were treated as on nominal scale. Only unweighted κ was computed for items with answer categories on nominal scale (e.g. IN1a). Observations with missing values for a given item in both questionnaires were excluded for calculating unweighted κ. Observed agreement (%) is also shown to regard the dependence of κ from the distribution of data [5]. In case of weighted κ we adjusted percentage agreement using the specific weighting matrix applied for κ itself. Confidence intervals (95 %) for κ were calculated according to Reichenheim [6], using bias corrected bootstrap estimates. We computed Krippendorf’s α (nominal scale) using the R package “irr” (version 0.84) and Bennett’s *S* according to [7]. Global Χ^2^ test was used to compare observed and expected proportions. Statistical analyses were conducted with Microsoft Excel 2010 (Microsoft Corp), STATA 12 IC (StataCorp LP), R 3.1.0 (The R Foundation for Statistical Computing) and IBM SPSS Statistics (version 20).

## Results

The characteristics of the study populations are listed in Table 1.

**Table 1:** Characteristics of the study population

	Total sample *n* (%)	Subsample of reliability study *n* (%)
**Sex**		
Female	162 (49.7)	47 (53.4)
Male	163 (50.0)	41 (46.6)
Missing values	1 (0.3)	–
**Age**		
20–29 years	24 (7.4)	5 (5.7)
30–39 years	30 (9.2)	9 (10.2)
40–49 years	85 (26.1)	20 (22.7)
50–59 years	87 (26.7)	22 (25.0)
60–69 years	99 (30.4)	32 (36.4)
Missing values	1 (0.3)	–
School education^a^		
Low	51 (15.9)	18 (20.5)
Middle	118 (36.2)	35 (39.8)
High	151 (46.3)	34 (38.6)
Missing values	6 (1.8)	1 (1.1)
Net equivalent income^b^		
≤ 1500 €	82 (25.2)	25 (28.4)
1501–3000 €	153 (46.9)	43 (48.9)
> 3000 €	62 (19.0)	14 (15.9)
Missing values/not specified	29 (8.9)	6 (6.8)
Migration status^c^		
No migration background	263 (80.7)	78 (88.6)
Migration background	59 (18.1)	9 (10.2)
Missing values/not specified	4 (1.2)	1 (1.1)
Study regions		
Hamburg	161 (49.4)	–
Hanover	165 (50.6)	88 (100)

^a^Grouping of school graduation according to [3]^b^Household net income per month weighted by number of members ≥ 14 years or < 14 years [2]^c^Definition of migration status according to [1]

### Self-assessed vulnerability to infections

**Fig. 1:**
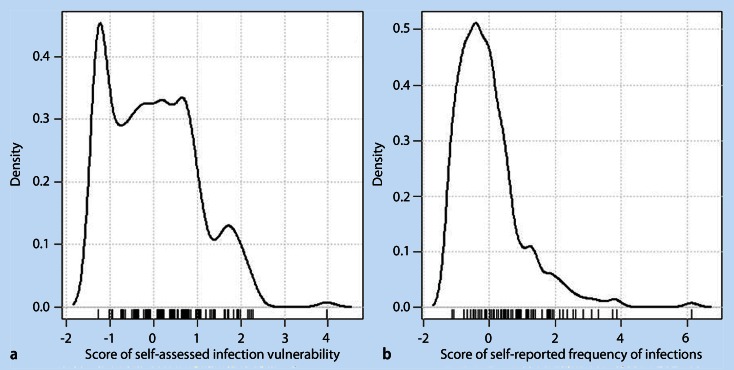
Distribution of **a** the self-assessed infection vulnerability score and **b** the score of self-reported frequency of infections. The scores were derived by using factor analysis (see the Methods section)

**Fig. 2:**
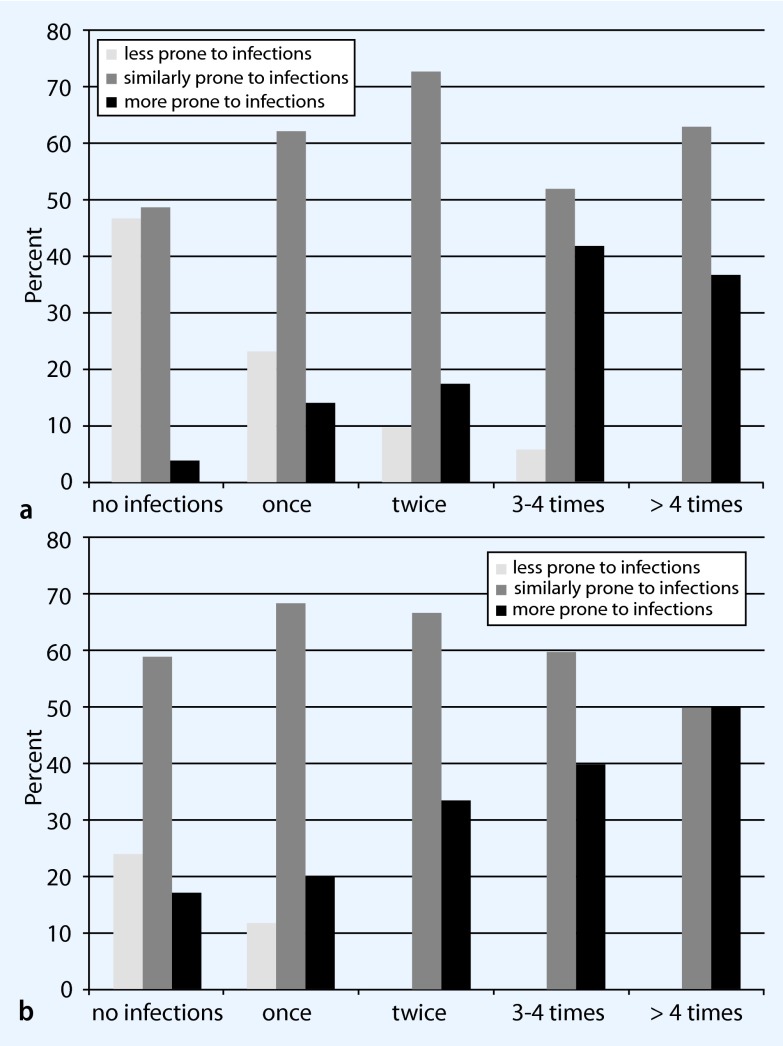
Relative self-reported frequency in the last 12 months of **a** upper respiratory tract and **b** gastrointestinal tract infections according to the three groups of self-assessed infection vulnerability

The evaluation of the internal consistency of the five-item instrument of self-assessed vulnerability to infections yielded a Cronbach’s α of 0.78. Removal of each item one at a time resulted in a decrease of the measure (ranging between 0.72 and 0.76), indicating that each item contributed well to the topic. The factor analysis yielded evidence of one factor (based on Eigenvalues > 1, see distribution in Fig. 1a, where higher values of the score indicate higher vulnerability to infections). All participants who were classified as “less prone to infections” reported to have different infectious diseases far less frequently than their peers (Table 2, second column). The two other groups were more heterogeneous in terms of self-compared vulnerability (Table 2, third and fourth columns). However, about 20 % of the participants in the “more prone to infections” group stated in agreement with their classification to have infections of bladder or kidney more often than their peers. Similarly, about one third of participants in the same group stated to have infections of the upper respiratory tract more often than their peers. Participants in the “less prone to infections” group were less likely to report infections in the past 12 months [IN1–7; exemplified by upper respiratory tract (Fig. 2a) and gastrointestinal tract (Fig. 2b) infections] than participants in the “more prone to infections” group. The reported frequencies of infections by participants who were classified as “similarly prone to infections” covered all frequency categories (see Fig. 2). The Spearman’s correlation of the two scores (self-reported frequencies and self-assessed vulnerability) yielded a value of 0.50 (*p* < 0.0001).

**Table 2:** Variables used to create the score of self-assessed vulnerability to infections compared to peers^a^

Infections	Less prone to infections (*n* = 53) %	Similarly prone to infections (*n* = 181) %	More prone to infections (*n* = 58) %
Upper respiratory tract infections (IH1)			
Far less frequent	100	19.3	0
Less frequent	0	53.0	19.0
Approximately equally frequent	0	17.1	50.0
More often	0	8.8	22.4
Much more often	0	1.7	8.6
Lower respiratory tract infections (IH2)			
Far less frequent	100	39.8	1.7
Less frequent	0	45.3	25.9
Approximately equally frequent	0	12.2	58.6
More often	0	2.2	8.6
Much more often	0	0.6	5.2
Gastrointestinal tract infections (IH3)			
Far less frequent	100	32.0	6.9
Less frequent	0	48.6	25.9
Approximately equally frequent	0	14.4	51.7
More often	0	4.4	10.3
Much more often	0	0.6	5.2
Bladder or kidney infections (IH4)			
Far less frequent	100	52.5	3.4
Less frequent	0	43.1	22.4
Approximately equally frequent	0	3.3	53.4
More often	0	1.1	19.0
Much more often	0	0	1.7
Infections of skin and mucosa (IH5)			
Far less frequent	100	50.3	12.1
Less frequent	0	38.1	29.3
Approximately equally frequent	0	8.8	46.6
More often	0	2.2	8.6
Much more often	0	0.6	3.4

^a^The score was derived by using factor analysis (see Methods section)

### Reliability study

**Fig. 3:**
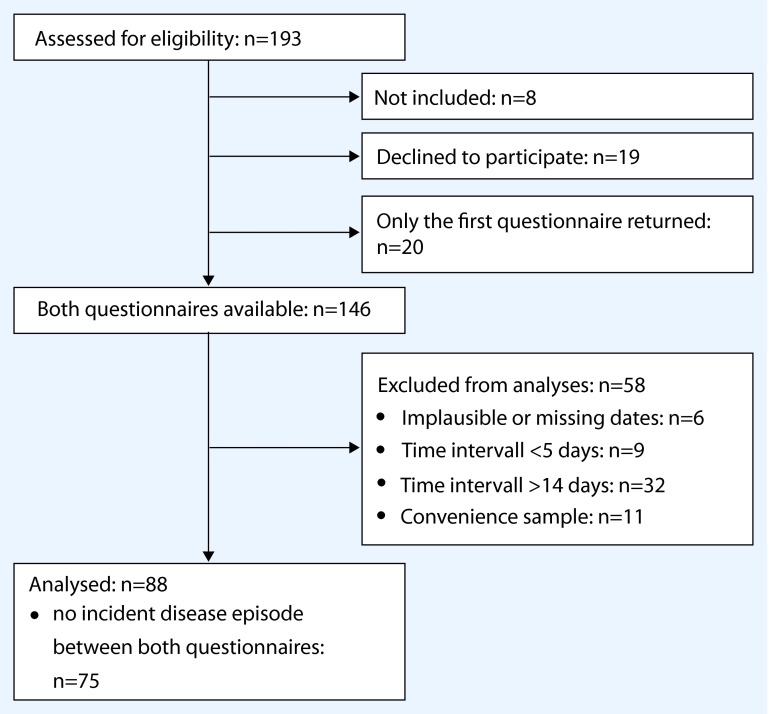
Flow chart of the reliability study

Of 146 participants for whom both questionnaires were available, 88 could be included in the analysis (Fig. 3). The proportions of men and women and the age distribution (10-year groups) did not depart significantly from the intended proportions within the GNC of 50 % (*p* = 0.18) or 10 %–10 %–26.7 %–26.7 %–26.7 % (see [8], *p* = 0.25), respectively.

Median duration of completing the first questionnaire was 7 min [interquartile range (IQR) 5–10, range 2–59, *n* = 87], the second questionnaire took 50 % of participants 5 min (IQR 4–7, range 2–40, *n* = 87). The median time interval between filling in both questionnaires was 8 days (IQR 7–10.5, *n* = 88). The weighted κ between answers of the first and the second questionnaire ranged between 0.65 and 0.87 for the items on infectious disease frequency in the last 12 months, for items on symptom occurrence in the past 12 months between 0.77 and 0.90, and for items on vulnerability compared to peers between 0.68 and 0.76. Antibiotic intake and influenza vaccination had κ values of 0.78 and 0.84, respectively. All percentage agreement, weighted/unweighted κ and Bennet’s *S* values are presented in Table 3. In addition to κ, the calculation of Krippendorf’s α for data on a nominal scale yielded identical values as the non-weighted κ (data not shown).

**Table 3:** Agreement of questionnaire one and two with weighted κ values for ordinal scales answers and unweighted if answers are on or interpreted as nominal scale (inclusion of “I don’t know” and missing values). Participants with a relevant disease episode between questionnaire one and two are excluded for disease- and symptom-based items as well as antibiotic intake

Item	Answer categories on ordinal scale (0– “ 4 times”)			Answer categories on or interpreted as nominal scale (incl. “Don’t know” and missings)		
	*n*	Linear weighted agreement [%]	Linear weighted Kappa and 95 % - confidence interval	*n*	Non-weighted agreement [%] (Bennet’s *S*)	Non-weighted k and 95 % - confidence interval
Disease-based items^a^						
URT infections (IN1)	72	92.7	0.77 [0.67–0.86]	75	73.3 (0.69)	0.65 [0.53–0.77]
Visit to GP or outpatient department (IN1a)	–	–	–	74	90.5 (0.87)	0.75 [0.62–0.90]
Hospitalisation (IN1b)	–	–	–	74	97.3 (0.96)	0 [n.c.]
LRT infections (IN2)	73	97.9	0.84 [0.67–0.92]	75	89.3 (0.88)	0.70 [0.49–0.83]
Visit to GP or outpatient department (IN2a)	–	–	–	73	87.7 (0.84	0.50 [0.18–0.75]
Hospitalisation (IN2b)	–	–	–	73	89.0 (0.85)	0.16 [-0.01–0.37]
GIT infections (IN3)	72	93.7	0.73 [0.57–0.87]	75	76.0 (0.72)	0.59 [0.46–0.75]
Visit to GP or outpatient department (IN3a)	–	–	–	73	91.8 (0.89)	0.57 [0.22–0.83]
Hospitalisation (IN3b)	–	–	–	73	94.5 (0.93)	−0.02 [-0.06–0]
Lip herpes (IN4)	73	98.6	0.87 [0.69–0.97]	75	92.0 (0.91)	0.75 [0.58–0.90]
Infections of skin or mucosa (IN5)	73	98.3	0.65 [0.20–0.94]	75	93.3 (0.92)	0.42 [0.09–0.65]
Bladder infection (IN6)	73	99.0	0.83 [0.69–0.93]	75	94.7 (0.94)	0.74 [0.50–0.89]
Kidney infection (IN7)	72	99.7	0.86 [0.85–0.86]	74	95.9 (0.95)	0.24 [0.16–0.40]
Symptom-based items^a^						
Cough (F1)	72	97.2	0.77 [0.52–0.95]	75	89.3 (0.88)	0.67 [0.47–0.84]
Fever (F2)	71	98.2	0.77 [0.60–0.93]	75	88.0 (0.86)	0.59 [0.44–0.78]
Diarrhoea (F3)	72	97.2	0.90 [0.81–0.96]	75	86.7 (0.84)	0.77 [0.65–0.89]
Self-assessed comparison to peers						
URT infections (IH1)	86	91.0	0.68 [0.57–0.79]	87	65.5 (0.59)	0.52 [0.39–0.67]
LRT infections (IH2)	87	94.0	0.74 [0.62–0.84]	87	78.2 (0.74)	0.67 [0.55–0.79]
GIT infections (IH3)	87	92.5	0.70 [0.57–0.80]	87	71.3 (0.66)	0.58 [0.42–0.71]
Bladder and kidney infection (IH4)	85	94.7	0.77 [0.62–0.86]	87	80.5 (0.77)	0.69 [0.56–0.81]
Infections of skin or mucosa (IH5)	87	94.0	0.69 [0.52–0.81]	87	80.5 (0.77)	0.66 [0.49–0.77]
Other						
Use of antibiotics^a^ (A1)	72	96.2	0.78 [0.66–0.89]	74	83.8 (0.81)	0.70 [0.55–0.84]
Influenza vaccination (V1)	–	–	–	88	92.0 (0.89)	0.84 [0.73–0.93]

*URT* upper respiratory tract; *LRT* lower respiratory tract; *GIT* gastrointestinal tract; *n.c.* cannot be calculated

^a^Only participants with no infectious disease episode between both questionnaires are included

## Discussion

### Self-assessed vulnerability to infections

We evaluated questions on self-assessed vulnerability to infections and demonstrated that they were reliable; the instrument also showed a high internal consistency. Furthermore, we observed a moderate correlation between self-reported frequency of infections and self-assessed vulnerability to infections. Research has shown that self-assessment of health status may be a valid measure of a respondent’s objective health status. For example, a single-item global self-rated health measure is a widely used instrument and has found application in many studies [9–11]. Advantages such as simplicity and ease of administration explain this broad usage of such instruments. The above mentioned single-item measure of global self-rated health has also been shown to predict morbidity and mortality [12, 13]. To our knowledge, no studies have examined questions on self-assessed vulnerability with regard to common infections/infectious diseases. The five-item instrument on self-assessed vulnerability to infections seems to be a promising instrument. However, further evaluation of self-assessed infections is required to validate this instrument. For example, self-assessed vulnerability to infections presented here might reflect not only comparative susceptibility but also psychosocial conditions. Self-assessment of infection vulnerability may be influenced by subjective factors. Thus, this evaluation of infection vulnerability based on self-assessed questions alone should be treated with caution.

### Reliability

We conducted a test–retest reliability study on an infectious disease questionnaire in Pretest 2 of the GNC in the Study Centre Hanover. Eighty-eight of Pretest 2 participants (random sample) could be included in our analysis. Cohen’s κ was calculated as the primary measure of agreement. To account for sampling, 95 % confidence intervals based on bootstrap estimation are shown indicating some relevant uncertainty of point estimates for a number of items (e.g. skin infections).

The short median duration of 7 or 5 min, respectively, for filling in the questionnaire supports the feasibility of its application. A process of habituation and learning cannot be ruled out since filling it in for the second time was generally faster. Non-transparent behavior like having a copy of the first questionnaire at home and using it as reference might influence duration and reliability measures as well. Yet in principle, the time interval in our study between the two copies of the questionnaire should suffice to avoid memory effects in order to ensure independent ratings on one hand and to guarantee stability of evaluated items on the other hand.

Our test–retest reliability study indicates that in general the questionnaire on self-reported infectious diseases is reliable. In order to evaluate agreement based on calculation of κ, the following categories are often used [4]: ≤ 0 (two items) poor, 0.01–0.2 slight (one item), 0.21–0.4 fair (no item), 0.41–0.60 moderate (two items), 0.61–0.80 substantial (12 items), and 0.81–1 almost perfect agreement (six items), choosing the higher κ if two are available for a given item.

Since linear weighting leads to more conservative κ estimates than quadratic weighting [14], the presented values for κ might underestimate agreement of questionnaire one and two on ordinal scales. By excluding observations with missing values for a given item in *both* questionnaires for calculating unweighted, i.e. nominal, κ this might underestimate the true agreement beyond chance as well.

κ depends not only on subjects’ agreement per se but also on frequency of categories and distribution of agreement and disagreement [4]. These characteristics mean that interpreting κ is not straight forward [4, 15]. To contextualise κ, percentage agreement is reported as well. This makes it possible to account for situations in which κ is low despite high percentage agreement due to the distribution of classifications, a situation called one of the intrinsic paradoxes of κ [4]. Problems due to distribution of marginals are generally part of our data since most mentioned diseases/symptoms/conditions do not occur evenly distributed over answer categories in the population. This might particularly apply to IN1b (hospitalisation for URT infection, κ = 0, percentage agreement 97.3 %), IN2b (hospitalisation for LRT infection, κ = 0.16, percentage agreement 89.0 %), and IN3b (hospitalisation for GIT infection κ = −0.02, percentage agreement 94.5 %), explaining the discrepancy between high percentage agreement and poor κ, and indicating that κ might be spuriously low in these cases. For IN2b, κ increases from 0.16 to 1.0 and from 89 % crude agreement to 100 % if observations with missing values for this item are excluded (left *n* = 65). This shows how slight changes in analysis strategy could influence κ substantially.

An alternative reliability coefficient, Krippendorf’s α, a very flexible measure of disagreement [16], adds no further information to the calculation of κ resulting in identical point estimates. A further addition to Cohen’s κ, Bennett’s *S*, can be regarded as generalisation of Byrt’s *prevalence-adjusted bias-adjusted k* (PABAK) [17]. Since *S* remodels the observed agreement [7] the results relate closely to the reported percentage agreement in Table 3 and adds to the notion of a spuriously low κ in the aforementioned cases.

In summary, despite a methodologically conservative approach the overall reliability of the infectious disease questionnaire using the answer categories on an ordinal scale or on a nominal scale and including “I don’t know” and missing values as separate categories can be interpreted as “moderate” to “very good” if κ and percentage agreement or Bennet’s S are both taken into account. Thus, measurement error and uncertainty of subjects’ own classification should be reasonably low.

## Conclusion

A five-item instrument on self-assessed vulnerability to infections seems to be promising. However, further evaluation of the instrument regarding, e.g. psychosocial influences, is needed. Thus, evaluation of infection vulnerability based on self-assessed questions alone should be treated with caution. The questionnaire on self-reported infectious diseases used in Pretest 2 of the GNC is a moderately reliable instrument and thus can be applied in future studies on infectious diseases.

Lessons learned for the main recruitment phase of the GNC:

A modified version of the infectious disease questionnaire will be used on Level 1 of the GNC.

## Electronic supplementary material

(pdf 136 kb)
