# Supplementary material for: Test–retest reliability of an infectious disease questionnaire and evaluation of self-assessed vulnerability to infections: Findings of Pretest 2 of the German National Cohort
Source: Bundesgesundheitsblatt Gesundheitsforschung Gesundheitsschutz. 2014 Oct 8;57(11):1300–7. doi: 10.1007/s00103-014-2045-x (PMC4210725; doi:10.1007/s00103-014-2045-x)
Supplement: Supplementary file 1 — (pdf 136 kb) [file 103_2014_2045_MOESM1_ESM.pdf]

ID-Nummer: ☐

<Für das Studienpersonal: Die ID-Nummer unbedingt auf jeder Seite in das vorgegebene Feld schreiben, drucken (Seriendruck) oder kleben>

**Start der Befragung**		
**S1**	Datum	☐☐ . ☐☐ . ☐☐☐☐<br>T T MM J JJJ
**S2**	Uhrzeit	☐☐ : ☐☐<br>Std. Min.
**IN0**	Sind Sie seit dem Ausfüllen des ersten Fragebogens zu Infektionskrankheiten vor ca. einer Woche **neu** an einer der folgenden Krankheiten (Infekt der Atemwege (inkl. Lunge)/des Magen-Darm-Trakts/der Niere oder Harnblase, Lippenherpes, Abszess oder Furunkel) erkrankt?<br><br>Ja ☐ Nein ☐ Weiß nicht ☐	

Infektionskrankheiten / Infektionen

Im folgenden Abschnitt (Fragen IN1 bis IN7) geht es um die <u>**Häufigkeit**</u> von unterschiedlichen Infektionen **in den vergangenen 12 Monaten**. Zählen Sie bitte zusammen, wie oft Sie in den vergangenen 12 Monaten an diesen Infektionen litten. Wenn Sie beispielsweise in den vergangenen 12 Monaten 3-mal Schnupfen und 1-mal Mittelohrinfektion hatten, dann sind es insgesamt 4 Infektionsepisoden (somit wählen Sie die Antwortmöglichkeit „3-4-mal“). Wenn Sie 5-mal Schnupfen hatten und keine anderen Infektionen der oberen Atemwege, dann wählen Sie die Antwortmöglichkeit „Mehr als 4-mal“.

Wichtig! Die Fragen IN1 bis IN7 beziehen sich auf die vergangenen 12 Monate.

IN1 Wie häufig hatten Sie <u>in den vergangenen 12 Monaten</u> eine Infektion der oberen Atemwege (z.B. Schnupfen, oder Infektionen von Nasennebenhöhlen, Mandeln, Mittelohr, Rachen, Kehlkopf)?

Keine	1-mal	2-mal	3-4-mal	Mehr als 4-mal	Weiß nicht
☐	☐	☐	☐	☐	☐

IN1a Waren Sie wegen mindestens einer dieser Infektionen in **ambulanter** ärztlicher Behandlung (Praxis oder Klinik)?

Ja ☐ Nein ☐ Weiß nicht ☐

IN1b Wurden Sie wegen mindestens einer dieser Infektionen **stationär** behandelt (d.h. mindestens eine Nacht im Krankenhaus)?

Ja ☐ Nein ☐ Weiß nicht ☐

ID-Nummer:

<Für das Studienpersonal: Die ID-Nummer unbedingt auf jeder Seite in das vorgegebene Feld schreiben, drucken (Seriendruck) oder kleben>

IN2 Wie häufig hatten Sie in den vergangenen 12 Monaten eine Infektion der unteren Atemwege (z.B. Bronchitis oder Lungenentzündung)?

Keine	1-mal	2-mal	3-4-mal	Mehr als 4-mal	Weiß nicht
☐	☐	☐	☐	☐	☐

IN2a Waren Sie wegen mindestens einer dieser Infektionen in **ambulanter** ärztlicher Behandlung (Praxis oder Klinik)?

Ja ☐ Nein ☐ Weiß nicht ☐

IN2b Wurden Sie wegen mindestens einer dieser Infektionen **stationär** behandelt (d.h. mindestens eine Nacht im Krankenhaus)?

Ja ☐ Nein ☐ Weiß nicht ☐

IN3 Wie häufig hatten Sie in den vergangenen 12 Monaten eine Infektion des Magen-Darm-Traktes (Durchfall mit oder ohne Erbrechen, auch bekannt als „Magen-Darm-Grippe)?

Keine	1-mal	2-mal	3-4-mal	Mehr als 4-mal	Weiß nicht
☐	☐	☐	☐	☐	☐

IN3a Waren Sie wegen mindestens einer dieser Infektionen in **ambulanter** ärztlicher Behandlung (Praxis oder Klinik)?

Ja ☐ Nein ☐ Weiß nicht ☐

IN3b Wurden Sie wegen mindestens einer dieser Infektionen **stationär** behandelt (d.h. mindestens eine Nacht im Krankenhaus)?

Ja ☐ Nein ☐ Weiß nicht ☐

IN4 Wie häufig hatten Sie in den vergangenen 12 Monaten Lippenherpes?

Keine	1-mal	2-mal	3-4-mal	Mehr als 4-mal	Weiß nicht
☐	☐	☐	☐	☐	☐

IN5 Wie häufig hatten Sie in den vergangenen 12 Monaten eine der folgenden Haut- oder Schleimhautinfektionen?

Furunkel oder Abszess (einfache „Pickel“ nicht zählen)

Keine	1-mal	2-mal	3-4-mal	Mehr als 4-mal	Weiß nicht
☐	☐	☐	☐	☐	☐

IN6 Wie häufig hatten Sie in den vergangenen 12 Monaten eine Infektion der Harnblase („Blasenentzündung“)?

Keine	1-mal	2-mal	3-4-mal	Mehr als 4-mal	Weiß nicht
☐	☐	☐	☐	☐	☐

ID-Nummer:

<Für das Studienpersonal: Die ID-Nummer unbedingt auf jeder Seite in das vorgegebene Feld schreiben, drucken (Seriendruck) oder kleben>

IN7 Wie häufig hatten Sie in den vergangenen 12 Monaten eine Infektion („Entzündung“) der Niere oder des Nierenbeckens?

Keine	1-mal	2-mal	3-4-mal	Mehr als 4-mal	Weiß nicht
☐	☐	☐	☐	☐	☐

Infekthäufigkeit

IH1 Verglichen mit anderen Menschen in meiner Altersgruppe leide ich an akuten Infekten der Atemwege (z.B. Schnupfen, oder Infektionen von Nasennebenhöhlen, Mandeln, Mittelohr, Rachen, Kehlkopf)…

Viel seltener	Seltener	Etwa gleich oft	Öfter	Viel öfter
☐	☐	☐	☐	☐

IH2 Verglichen mit anderen Menschen in meiner Altersgruppe leide ich an akuten Infekten der unteren Atemwege (Bronchitis oder Lungenentzündung)…

Viel seltener	Seltener	Etwa gleich oft	Öfter	Viel öfter
☐	☐	☐	☐	☐

IH3 Verglichen mit anderen Menschen in meiner Altersgruppe leide ich an akuten Infekten des Verdauungstraktes (Durchfall mit oder ohne Erbrechen)…

Viel seltener	Seltener	Etwa gleich oft	Öfter	Viel öfter
☐	☐	☐	☐	☐

IH4 Verglichen mit anderen Frauen [*Männern*] in meiner Altersgruppe leide ich an akuten Infektionen der Harnblase, Niere oder Nierenbecken…

Viel seltener	Seltener	Etwa gleich oft	Öfter	Viel öfter
☐	☐	☐	☐	☐

IH5 Verglichen mit anderen Menschen in meiner Altersgruppe leide ich an Infektionen der Haut (z.B. Furunkel oder Abszess. Einfache „Pickel“ nicht zählen)….

Viel seltener	Seltener	Etwa gleich oft	Öfter	Viel öfter
☐	☐	☐	☐	☐

Antibiotikaeinnahme

A1 Wie häufig hat Ihnen in den vergangenen 12 Monaten ein Arzt Antibiotika verschrieben?

Auch zählen wenn Sie das Medikament nicht eingenommen haben!

Salben oder andere äußere Anwendungen nicht mitzählen.

Keine	1-mal	2-mal	3-4-mal	Mehr als 4-mal	Weiß nicht
☐	☐	☐	☐	☐	☐

ID-Nummer:

<Für das Studienpersonal: Die ID-Nummer unbedingt auf jeder Seite in das vorgegebene Feld schreiben, drucken (Seriendruck) oder kleben>

Anmerkung! Die Fragen F1 bis F3 beziehen sich auf die vergangenen 12 Monate.

F1 Wie häufig hatten Sie in den vergangenen 12 Monaten Husten, der länger als 4 Wochen dauerte?

Keine	1-mal	2-mal	3-4-mal	Mehr als 4-mal	Weiß nicht
☐	☐	☐	☐	☐	☐

F2 Wie häufig hatten Sie in den vergangenen 12 Monaten Fieber (>38°C)? (wenn das Fieber länger als 1 Tag dauerte, bitte als eine Episode zählen; z.B. Fieber für 7 Tage = 1-mal)

Keine	1-mal	2-mal	3-4-mal	Mehr als 4-mal	Weiß nicht
☐	☐	☐	☐	☐	☐

F3 Wie häufig hatten Sie in den vergangenen 12 Monaten Durchfall? Durchfall ist definiert als mindestens 3-mal flüssiger/breiiger Stuhlgang innerhalb von 24 Stunden (wenn der Durchfall länger als 1 Tag dauerte, bitte als eine Episode zählen; z.B. Durchfall für 4 Tage = 1-mal)

Keine	1-mal	2-mal	3-4-mal	Mehr als 4-mal	Weiß nicht
☐	☐	☐	☐	☐	☐

Impfungen

**V1**	Wurden Sie **jemals** gegen Grippe (Influenza) geimpft?	☐<br>☐<br>☐	Ja<br>Nein ---------------------→ weiter zu Frage<br>Weiß nicht ---------------------→ weiter zu Frage	 **T1**<br>**T1**
**V2**	In welcher Regelmäßigkeit lassen Sie sich gegen Grippe impfen?	☐<br>☐<br>☐<br>☐<br>☐	Jedes Jahr; seit wann: Jahr: |___|___|___|___| Fast jedes Jahr; seit wann: Jahr: |___|___|___|___| Seltener als fast jedes Jahr<br>Bis jetzt nur einmal<br>Weiß nicht	
**V3**	Wann wurden Sie **zuletzt** gegen Grippe (Influenza) geimpft?	☐	Jahr: |___|___|___|___| oder<br>Weiß nicht	

ID-Nummer:

<Für das Studienpersonal: Die ID-Nummer unbedingt auf jeder Seite in das vorgegebene Feld schreiben, drucken (Seriendruck) oder kleben>

Tiere

T1 Hatten Sie **<u>jemals</u>** <u>über einen Zeitraum von mehr als sechs Monaten</u>, privat oder beruflich, regelmäßig (d.h. mehr als einmal pro Woche) Umgang mit Haustieren?

- ☐ Ja
- ☐ Nein ---------------------→ weiter zu Frage **T2**
- ☐ Weiß nicht ---------------→ weiter zu Frage **T2**

Wenn Ja, mit welchem Tier/mit welchen Tieren?

	Ja	Nein	Weiß nicht
Hund	☐	☐	☐
Katze	☐	☐	☐
Nagetier (z.B. Hamster, Kaninchen, Meerschweinchen, Maus, Ratte)	☐	☐	☐
Ziervogel (z.B. Wellensittich, Papagei, Taube)	☐	☐	☐
Reptilien (z.B. Schildkröten, Leguane, Schlangen)	☐	☐	☐

Andere Tiere, wenn ja, welche? …………………………………………..

T2 Leben in Ihrem Haushalt Haustiere?

- ☐ Ja
- ☐ Nein--------------------→ weiter zu Frage **S3**
- ☐ Weiß nicht------------→ weiter zu Frage **S3**

Wenn Ja, welches Tier/welche Tiere?

	Ja, ausschließlich in der Wohnung	Ja, in oder außerhalb der Wohnung	Wenn Ja, Anzahl	Nein	Weiß nicht
Hund	☐	☐	………..	☐	☐
Katze	☐	☐	………..	☐	☐
Nagetier (z.B. Hamster, Kaninchen, Meerschweinchen, Maus, Ratte)	☐	☐	………..	☐	☐
Ziervogel (z.B. Wellensittich, Papagei, Taube)	☐	☐	………..	☐	☐
Reptilien (z.B. Schildkröten, Leguane, Schlangen)	☐	☐	………..	☐	☐

Andere Tiere, wenn ja, welche? …………………………………………..

Ende der Befragung

S3 Uhrzeit Befragung Ende ☐☐ Std. ☐☐ Min.

Vielen Dank für Ihre Mitarbeit!
